# Supplementary material for: Revealing the three-dimensional arrangement of polar topology in nanoparticles
Source: Nat Commun. 2024 May 8;15:3887. doi: 10.1038/s41467-024-48082-x (PMC11078976; doi:10.1038/s41467-024-48082-x)
Supplement: Supplementary file 1 — Supplementary Information [file 41467_2024_48082_MOESM1_ESM.pdf]

Supplementary Information

for

Revealing the Three-Dimensional Arrangement of Polar Topology in Nanoparticles

Chaehwa Jeong[1], Juhyeok Lee[1,2,3], Hyesung Jo[1], Jaewhan Oh[1], Hionsuck Baik[4], Kyoung-June Go[5], Junwoo Son[6], Si-Young Choi[5,7], Sergey Prosandeev[8], Laurent Bellaiche[8] and Yongsoo Yang[1,9*]

[1] *Department of Physics, Korea Advanced Institute of Science and Technology (KAIST), Daejeon 34141, Republic of Korea*

[2] *Energy Geosciences Division, Lawrence Berkeley National Laboratory, Berkeley, CA 94720, USA*

[3] *National Center for Electron Microscopy, Molecular Foundry, Lawrence Berkeley National Laboratory, Berkeley, CA 94720, USA*

[4] *Korea Basic Science Institute (KBSI), Seoul 02841, Republic of Korea*

[5] *Department of Materials Science and Engineering, Pohang University of Science and Technology (POSTECH), Pohang 37673, Republic of Korea*

[6] *Department of Materials Science and Engineering, Research Institute of Advanced Materials, Seoul National University, Seoul 08826, Republic of Korea*

[7] *Center for Van der Waals Quantum Solids, Institute for Basic Science (IBS), Pohang 37673, Republic of Korea*

[8] *Physics Department and Institute for Nanoscience and Engineering, University of Arkansas, Fayetteville, Arkansas 72701, USA*

[9] *Graduate School of Semiconductor Technology, School of Electrical Engineering, Korea Advanced Institute of Science and Technology (KAIST), Daejeon 34141, Republic of Korea*

*Corresponding author, email: yongsoo.yang@kaist.ac.kr

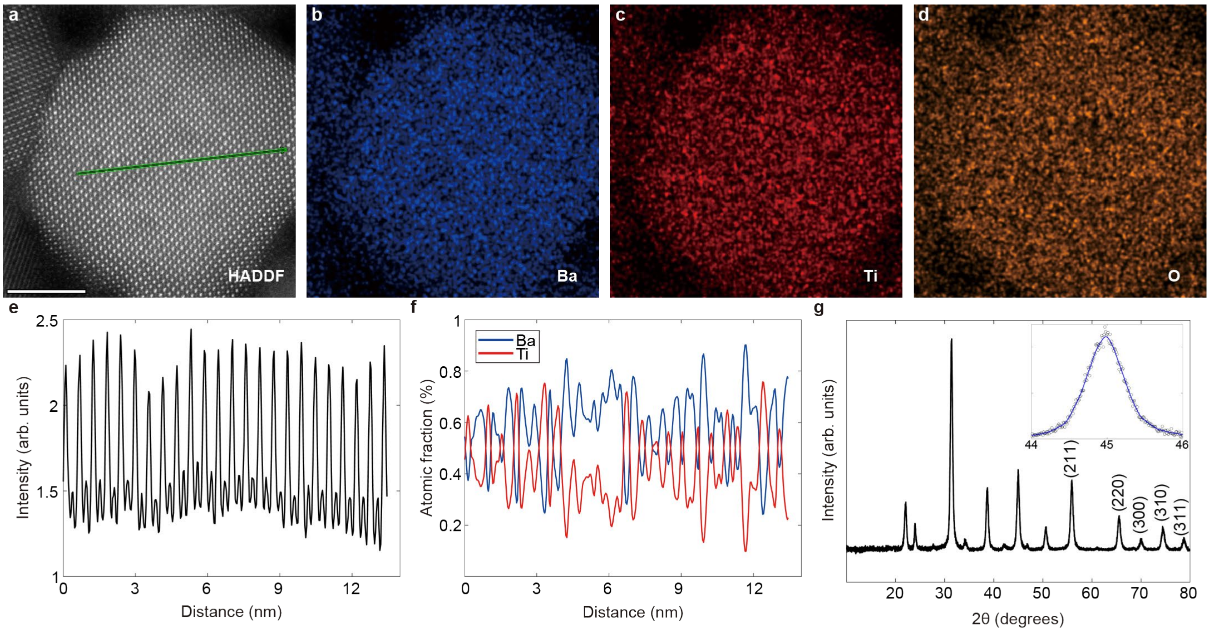

Supplementary Figure 1 | Characterization of a $BaTiO_3$ nanoparticle. **a**, An ADF-STEM image of a representative $BaTiO_3$ nanoparticle viewed along the <001> direction. Scale bar, 5 nm. **b-d**, The EDS intensity maps after Gaussian smoothing (standard deviations of 2 pixels) for Ba (**b**), Ti (**c**), and O (**d**). **e**, The ADF-STEM line scan profile within the region marked with a green box in (**a**); the alternating Ba and Ti columns can be clearly identified (weaker peaks represent the Ti columns). **f**, The atomic fraction line profile of Ba (blue) and Ti (red) within the region marked with the same green box in (**a**), confirming that the peaks in (**e**) (i.e., the atom columns) are indeed the alternating Ba and Ti columns. **g**, PXRD pattern of the $BaTiO_3$ nanoparticles. The five peaks used for the lattice constant determination are indexed based on the pseudocubic convention in the figure. The inset shows the PXRD pattern in the two-theta angular range of 44° to 46° corresponding to the (002) diffraction peak. The black dots represent the measured PXRD data and the blue solid lines indicate the measured data after Gaussian smoothing. Data of (**e-g**) are provided as a Supplementary Data 1 file.

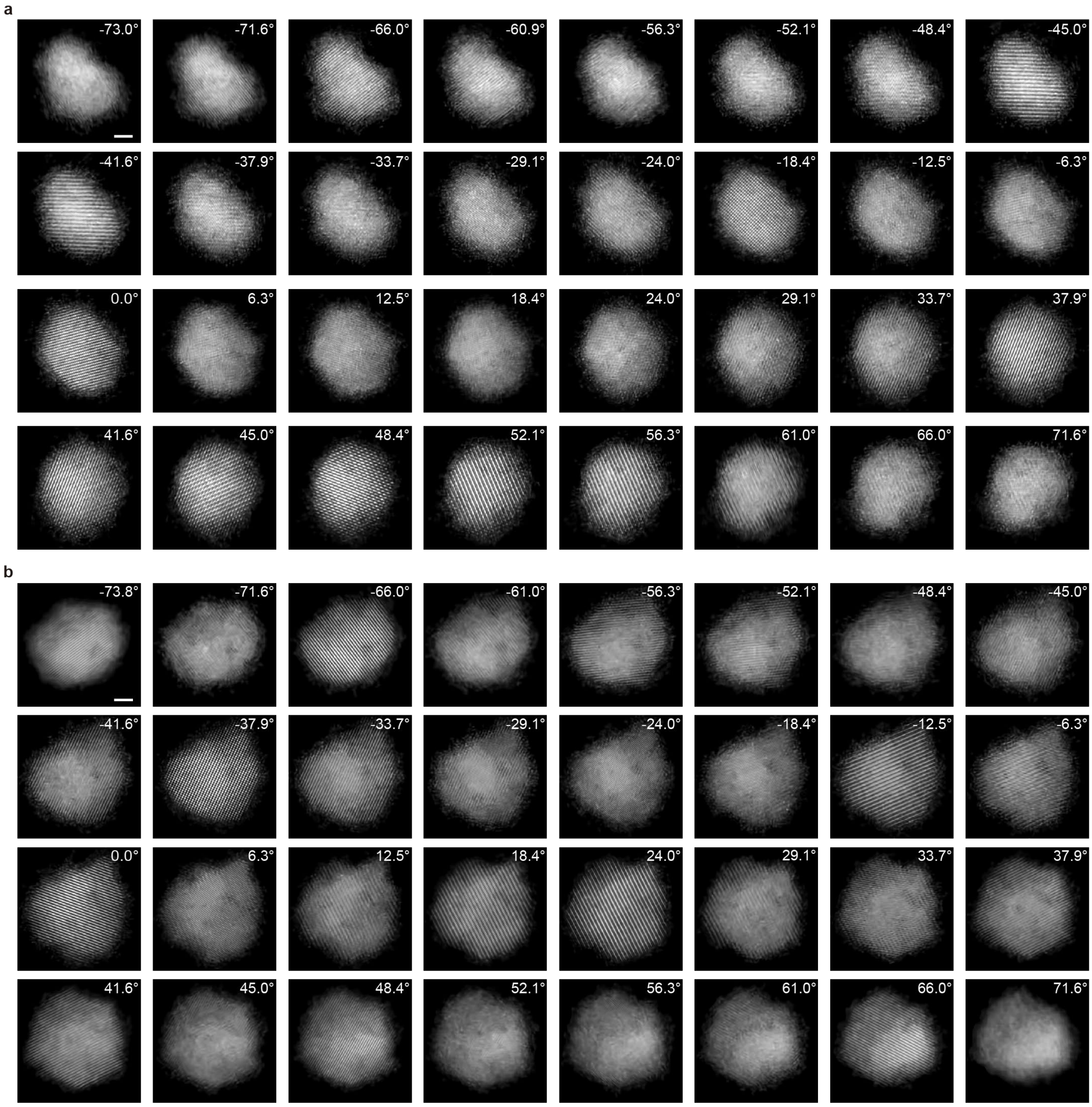

Supplementary Figure 2 | Experimental tomographic tilt series of the $BaTiO_3$ nanoparticles. a, **b**, 32 post-processed ADF-STEM images of the Particle 1 (8.8 nm) (**a**), and Particle 2 (10.1 nm) (**b**) $BaTiO_3$ nanoparticles. Tilt angles of Particle 1 and Particle 2 ranged from –73.0° to +71.6° and –73.8° to +71.6°, respectively. The total electron doses of the tilt series of Particle 1 and Particle 2 are 1.01×10^5 *e* $Å^{-2}$ and 9.52×10^4 *e* $Å^{-2}$, respectively. Scale bar, 2 nm.

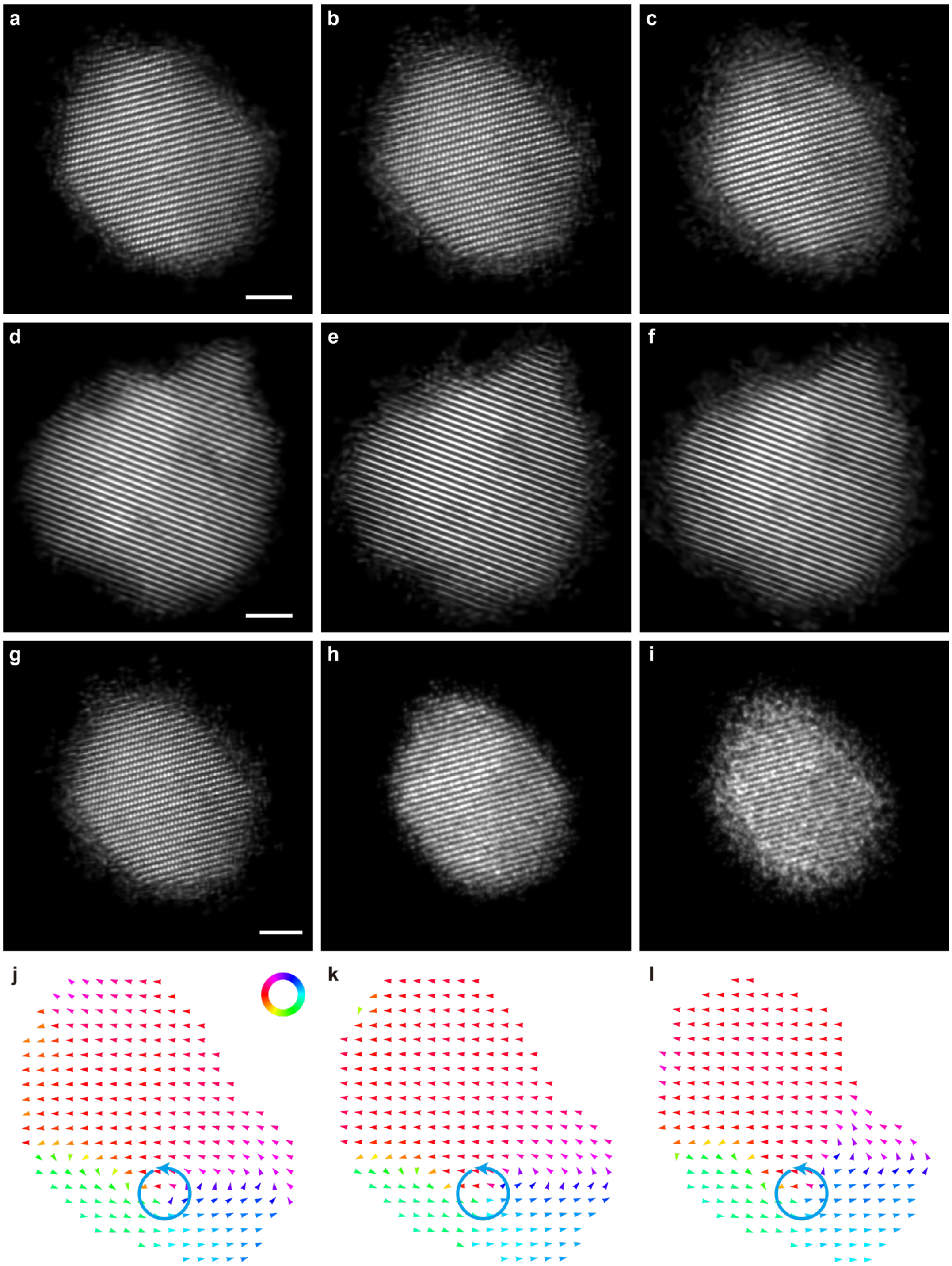

Supplementary Figure 3 | Comparison of the zero-degree projections during the experiments and simulation for the effect of surface diffusion during tilt series measurement. a-f, The ADF-STEM images at the zero-degree obtained at the beginning (**a**, **d**), in the middle (**b**, **e**), and at the end of the experiment (**c**, **f**) for Particle 1 (**a-c**) and Particle 2 (**d-f**), respectively. The zero-degree projections of (**b**) and (**e**) were used for the tomographic reconstructions, respectively. Scale bar, 2 nm. **g**, The ADF-STEM image at the zero-degree obtained in the middle of the tilt series acquisition for Particle 1. Scale bar, 2 nm. **h**, Linearly projected 3D potential volume obtained from the experimentally determined atomic structure of Particle 1 along the zero-degree direction. **i**, A linear projection image similar to (**h**), which is obtained from a 3D potential volume derived from the atomic structure where the surface atoms are randomly shifted with an RMSD of 300 pm (Methods). **j-l**, In-plane atomic displacement directions map of a Ti atomic layer from the atomic structure reconstructed from the experimental tilt series (**j**), a tilt series generated by linearly projecting the 3D potential volume of the atomic structure of Particle 1 (**k**), and a similarly obtained tilt series for which half of the projections are replaced with the linear projections from the structure affected by the random surface displacement (**l**) (Methods).

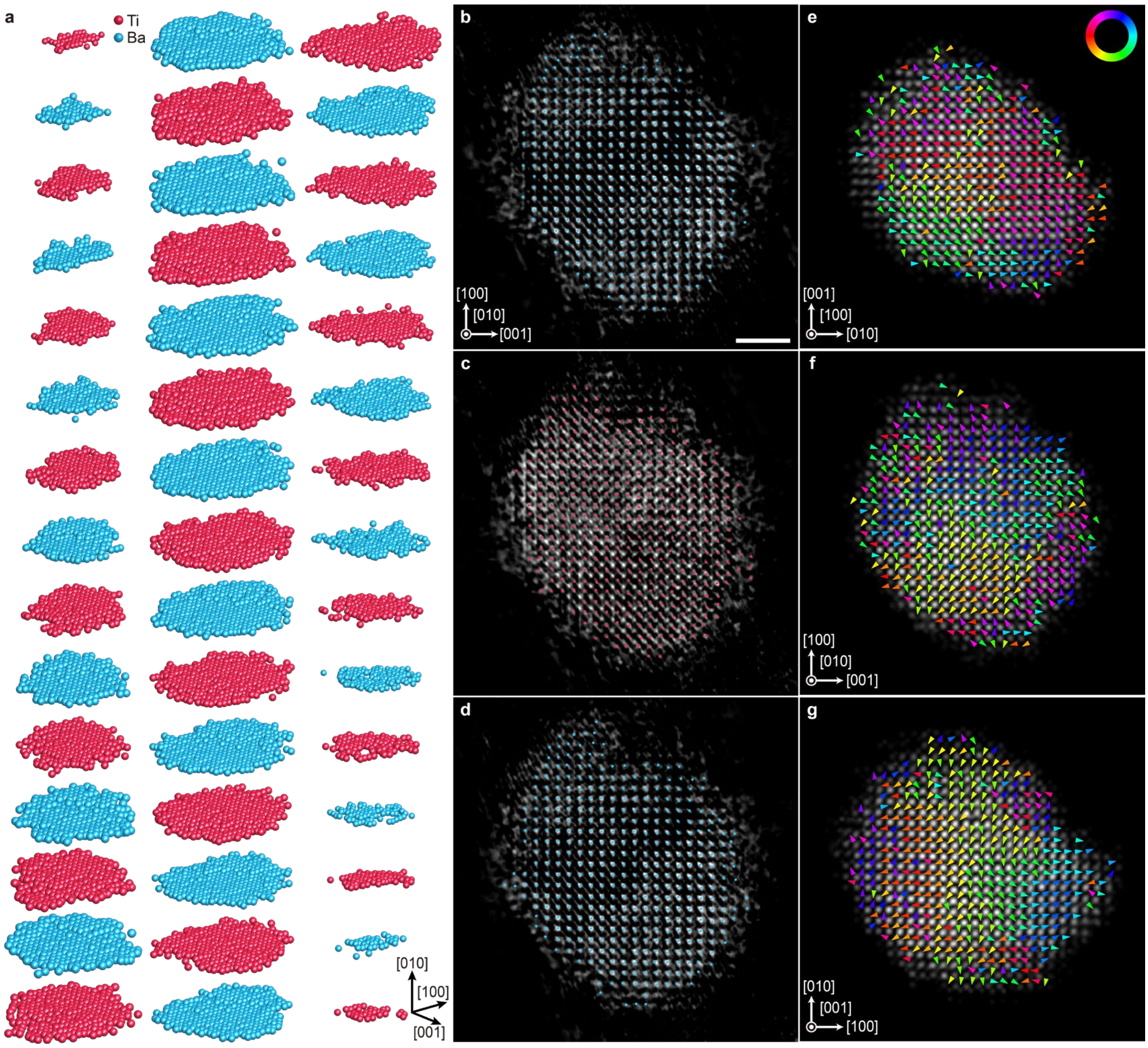

Supplementary Figure 4 | Determination of the 3D atomic structure of a 8.8 nm $BaTiO_3$ nanoparticle (Particle 1). a, Cation atomic layers along the [010] crystallographic direction, showing 3D atomic arrangements of alternating Ba and Ti layers. **b-d**, 1.07 Å thick internal slices of the 3D tomogram along [010] direction for three consecutive atomic layers near the core of the particle. The intensity of the sliced tomogram is depicted in grayscale, while the atomic coordinates of Ba and Ti are represented with blue and red dots, respectively. Scale bar, 2 nm. **e-g**, Linear projections of a 3D intensity volume representing the determined 3D atomic structure, projected along [100] (**e**), [010] (**f**), and [001] (**g**) directions, respectively. The grayscale background indicates the projected intensity, where Ba and Ti columns can be distinguished by their relative intensities (Ti columns show weaker contrasts). The arrows represent the Ti displacements, calculated as the deviation of the identified Ti column positions from the geometric centers of the four neighboring Ba column positions. The arrows are colored based on the displacement direction, as given in the color wheel in (**e**).

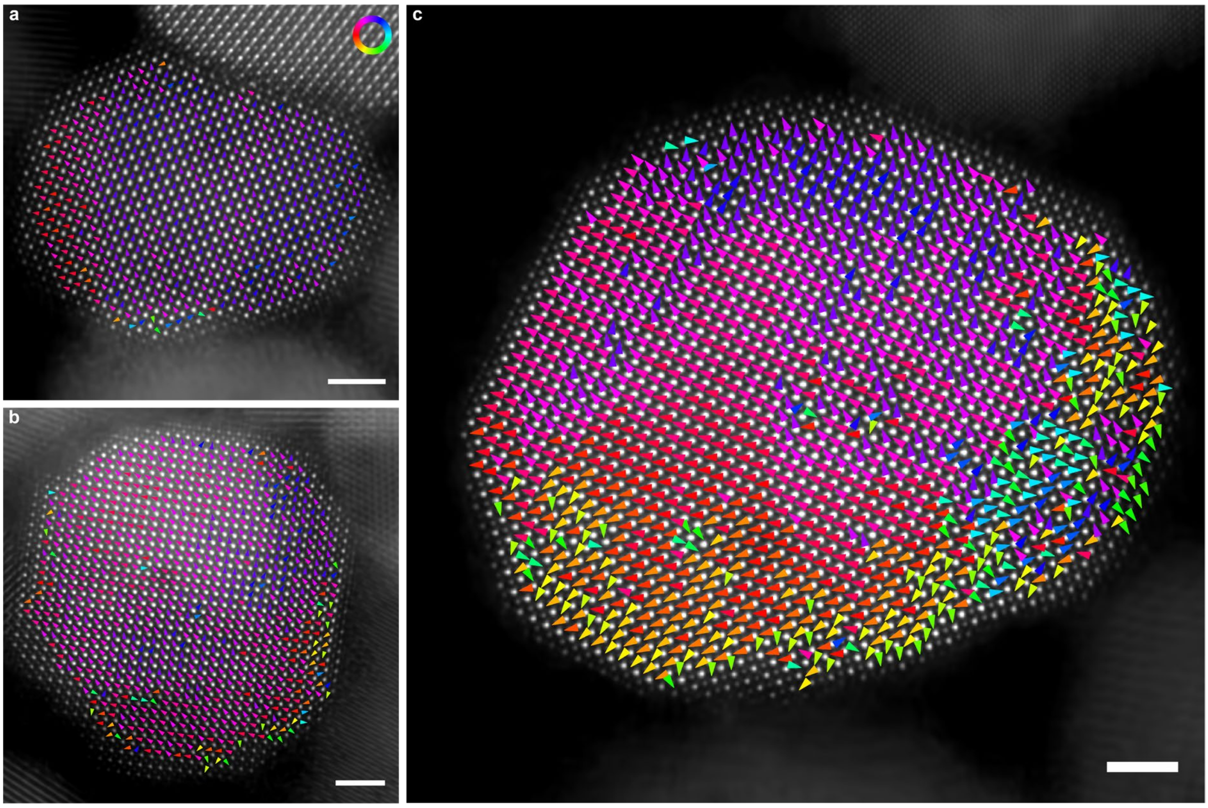

Supplementary Figure 5 | 2D Ti atomic displacement maps of $BaTiO_3$ nanoparticles. a-c, ADF-STEM images of <001> zone axes for three different $BaTiO_3$ nanoparticles, where the Ti displacement directions are overlaid. The projected sizes of the nanoparticles are estimated to be 9.6 × 12.2 nm^2 (**a**), 13.0 × 14.2 nm^2 (**b**), and 17.2 × 17.9 nm^2 (**c**). The arrows represent the directions of the Ti displacements calculated as the deviation of the identified Ti column positions from the geometric centers of the four neighboring Ba column positions. The arrows are colored based on the displacement direction as given in the color wheel in (**a**). For each nanoparticle, the majority of the displacements are aligned along the same direction, while some locally disordered or locally vortex-like features can be identified in some regions. Scale bar, 2 nm.

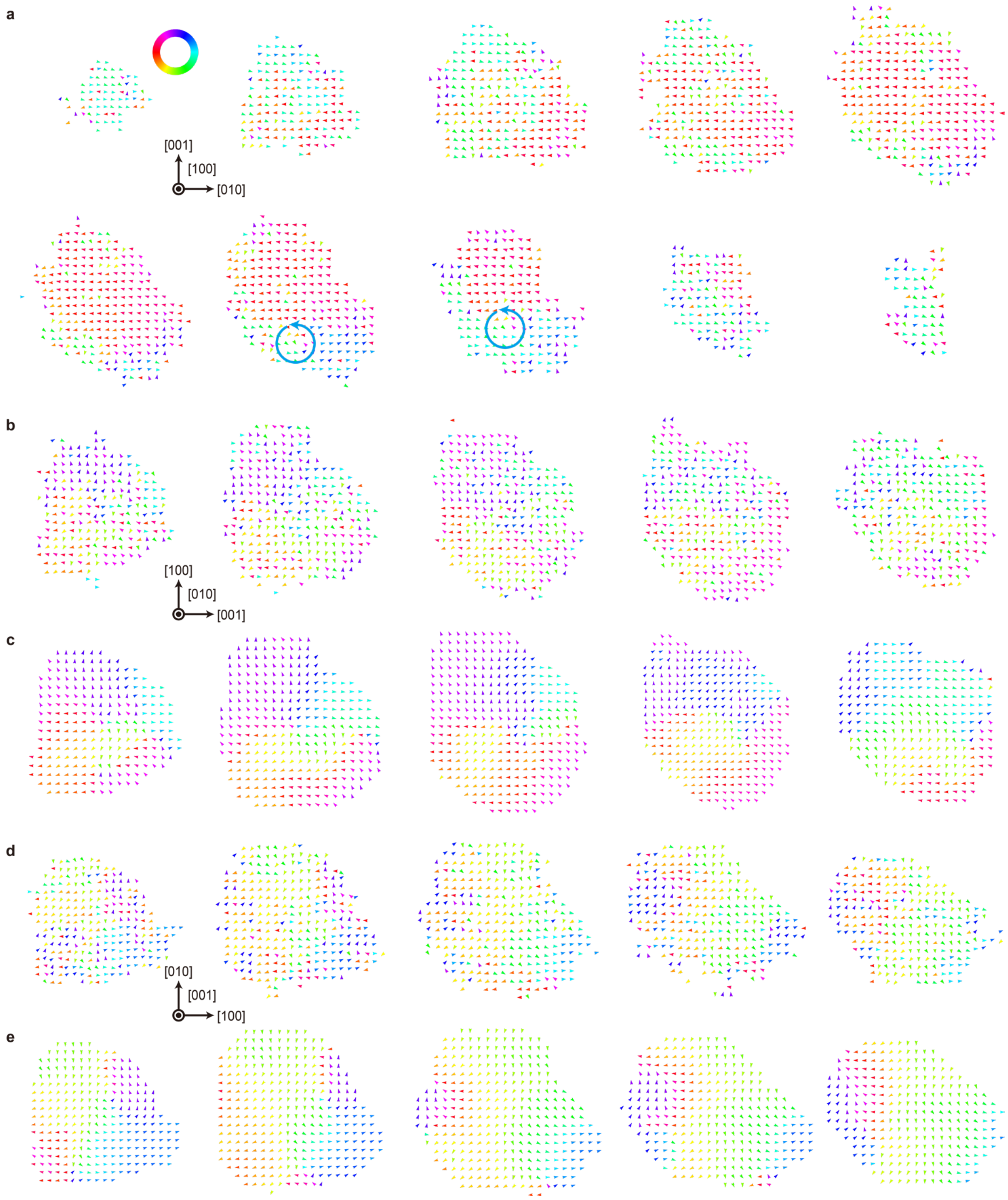

Supplementary Figure 6 | Sliced maps showing the 3D distribution of Ti atomic displacements for the 8.8 nm $BaTiO_3$ (Particle 1). a-e, In-plane atomic displacement direction maps of representative Ti atomic layers near the core of the particle sliced along the [100] (**a**), the [010] (**b**, **c**), and the [001] (**d**, **e**) direction before (**a**, **b**, **d**) and after (**c**, **e**) applying a Gaussian kernel. Note that the spacing between the plotted layers is 2 unit cells. The arrows are colored based on the displacement direction as given in the color wheel in (**a**). Even before applying the Gaussian kernel, a counterclockwise vortex along the [100] direction can be clearly identified (marked with blue arrows). The distance between the colored arrows is 3.75 Å.

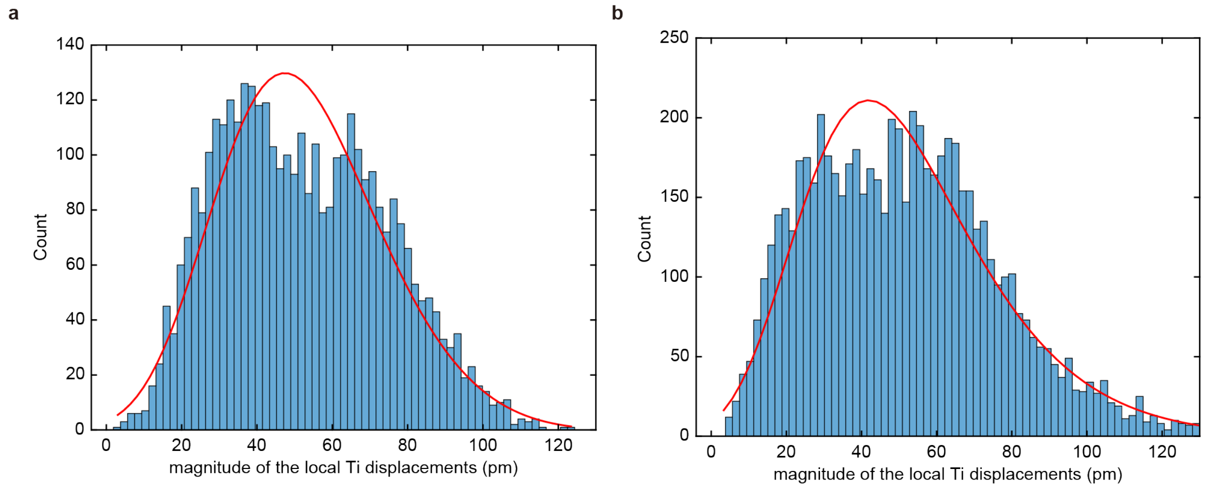

Supplementary Figure 7 | Distribution of the magnitude of the kernel-averaged local Ti atomic displacements. a-b, Histograms of the magnitude of the local Ti atomic displacement for Particle 1 (**a**), and Particle 2 (**b**), respectively. The red solid line represents the fitted generalized extreme value distribution, with the location parameter (μ) of the fitted distribution being 43.5 pm for Particle 1 and 40.5 pm for Particle 2, respectively. Note that the displacement vector at each atom position is interpolated from the kernel-averaged displacement vector map. Data of (**a-b**) are provided as a Supplementary Data 1 file.

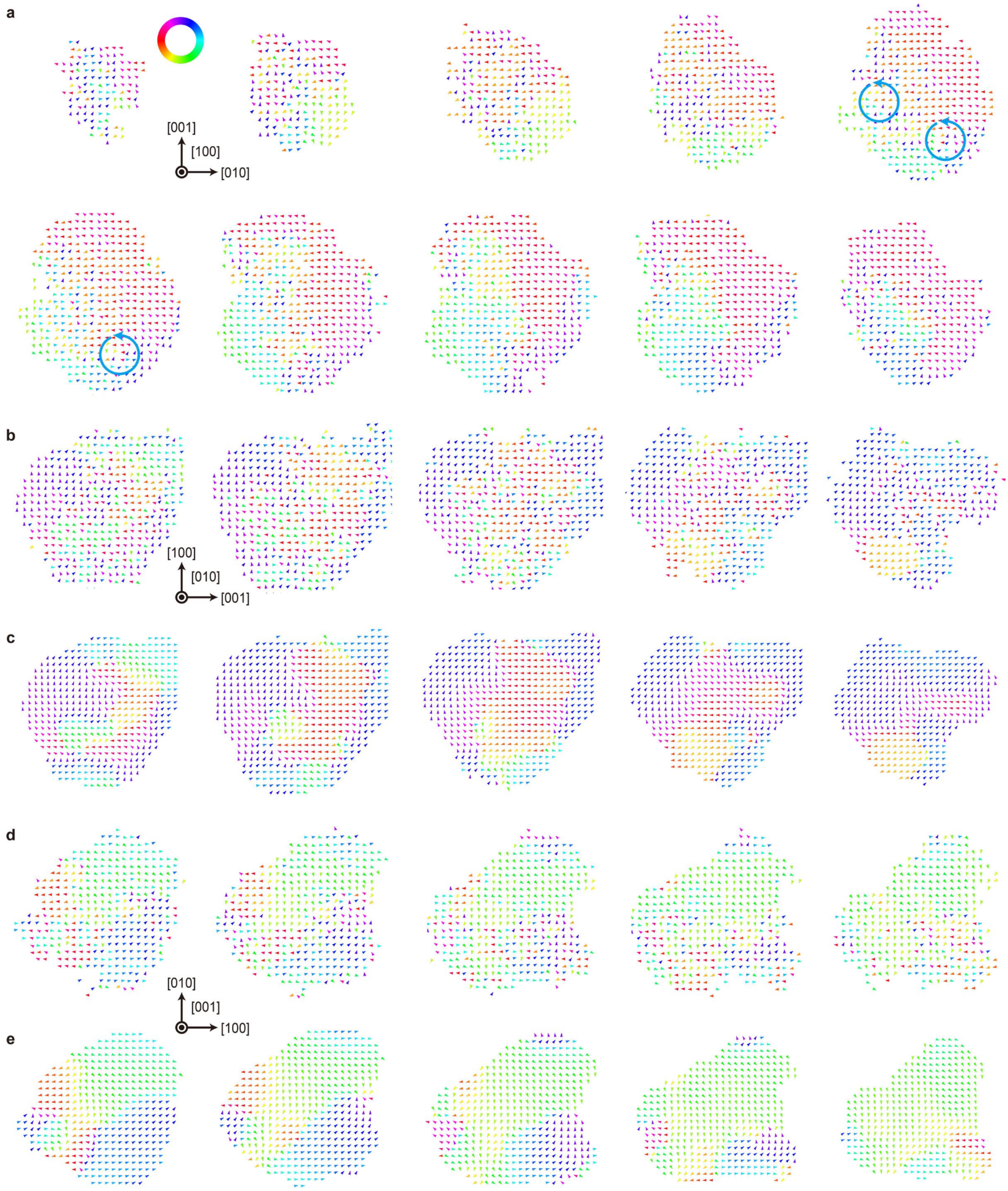

Supplementary Figure 8 | Sliced maps showing the 3D distribution of Ti atomic displacements for the 10.1 nm $BaTiO_3$ (Particle 2). a-e, In-plane atomic displacement direction maps of representative Ti atomic layers near the core of the particle sliced along the [100] (**a**), the [010] (**b, c**), and the [001] (**d, e**) direction before (**a, b, d**) and after (**c, e**) applying a Gaussian kernel. Note that the spacing between the plotted layers is 2 unit cells. The arrows are colored based on the displacement direction as given in the color wheel in (**e**). Even before applying a Gaussian kernel, multiple counterclockwise vortices along the [100] direction can be clearly seen (marked with blue arrows). The distance between the colored arrows is 3.75 Å.

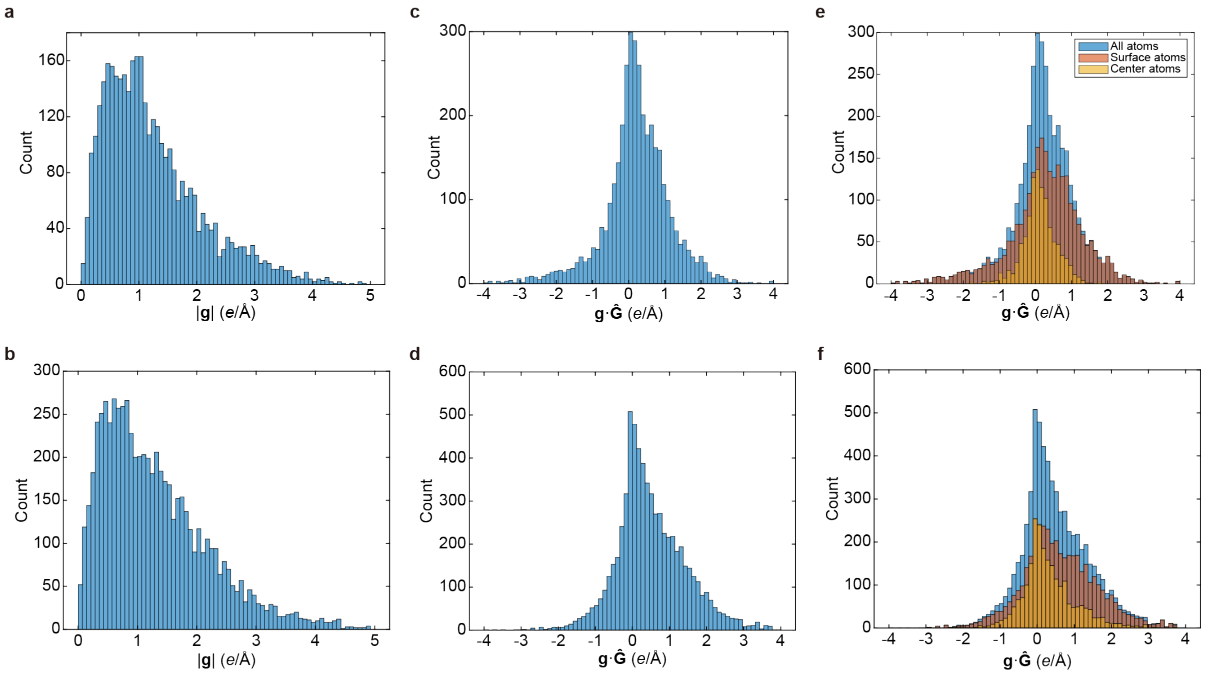

Supplementary Figure 9 | Distribution of local toroidal moments. a, **b**, Histograms of the absolute value of the local toroidal moment **g** for the Particle 1 (**a**), and the Particle 2 (**b**), respectively. **c**, **d**, Histograms of the vector components of the local toroidal moment **g** along the direction of averaged toroidal moment ($\widehat{\mathbf{G}}$) for the Particle 1 (**c**), and the Particle 2 (**d**), respectively. **e**, **f**, Histograms of the vector components of the local toroidal moment **g** along the direction of averaged toroidal moment ($\widehat{\mathbf{G}}$) for all atoms (blue), surface atoms (red), and core atoms (yellow) of the Particle 1 (**e**), and the Particle 2 (**f**), respectively. The surface atoms were determined by applying the Alpha-shape algorithm with a shrink factor of 1.0 (atoms within approximately 3 pseudocubic unit cells from the surface were selected as the surface atoms). The number of surface Ti atoms was 2691 (Particle 1) and 4307 (Particle 2), respectively. The average of $\mathbf{g} \cdot \widehat{\mathbf{G}}$ for all atoms, surface atoms, and core atoms were 0.22, 0.26, and 0.11 e Å^{-1} for Particle 1, and 0.51, 0.61, and 0.32 e Å^{-1} for Particle 2, respectively. Data of (**a-f**) are provided as a Supplementary Data 1 file.

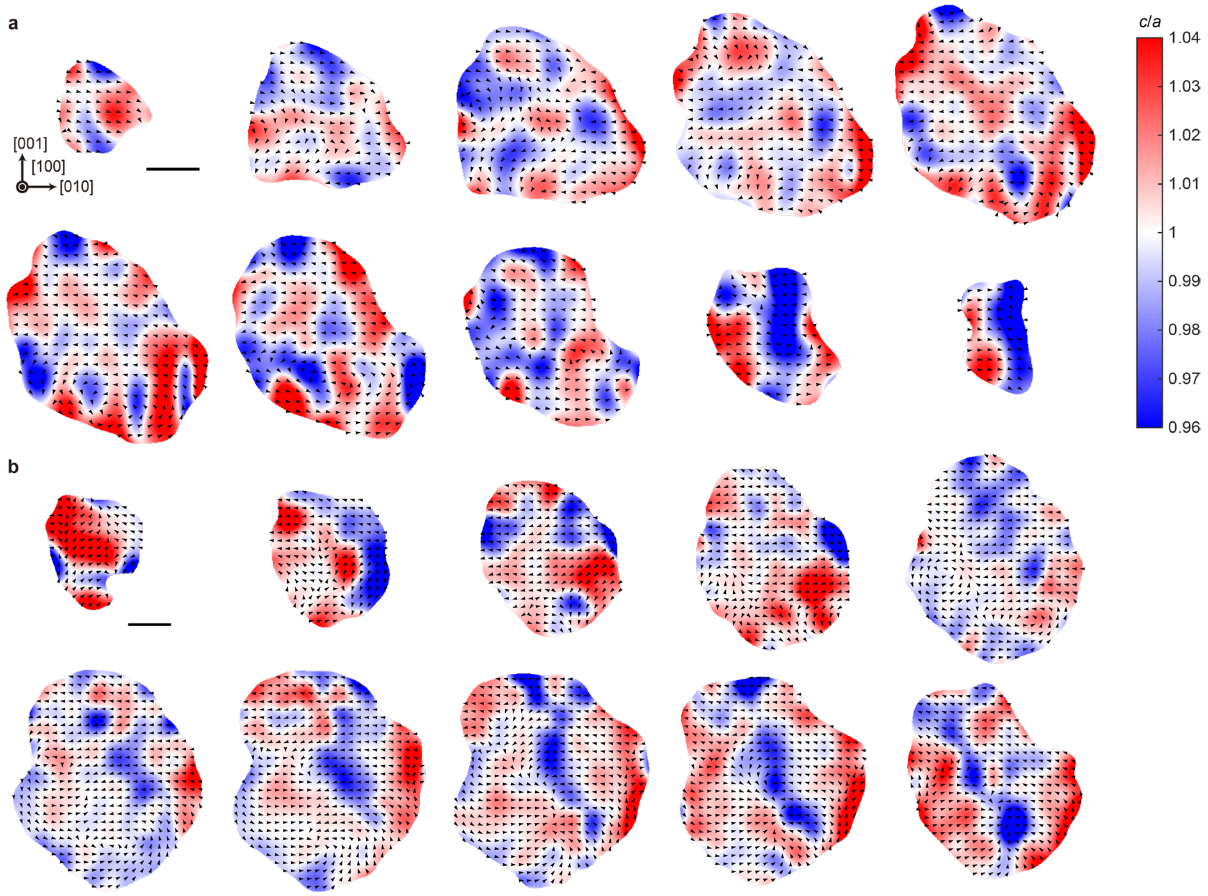

Supplementary Figure 10 | Representative sliced maps showing the tetragonality map. a, **b**, Tetragonality (*c*/*a*) maps of representative Ti atomic layers sliced along the [100] direction of the Particle 1 (8.8 nm) (**a**), and Particle 2 (10.1 nm) (**b**). The in-plane directions of the Ti displacements are overlaid (black arrows). Note that the blue and red colors represent the local *c*/*a* ratio. The precisions of the local *c*/*a* ratios are 0.012 for Particle 1 and 0.011 for Particle 2, determined through standard error propagation. Scale bar, 2 nm.

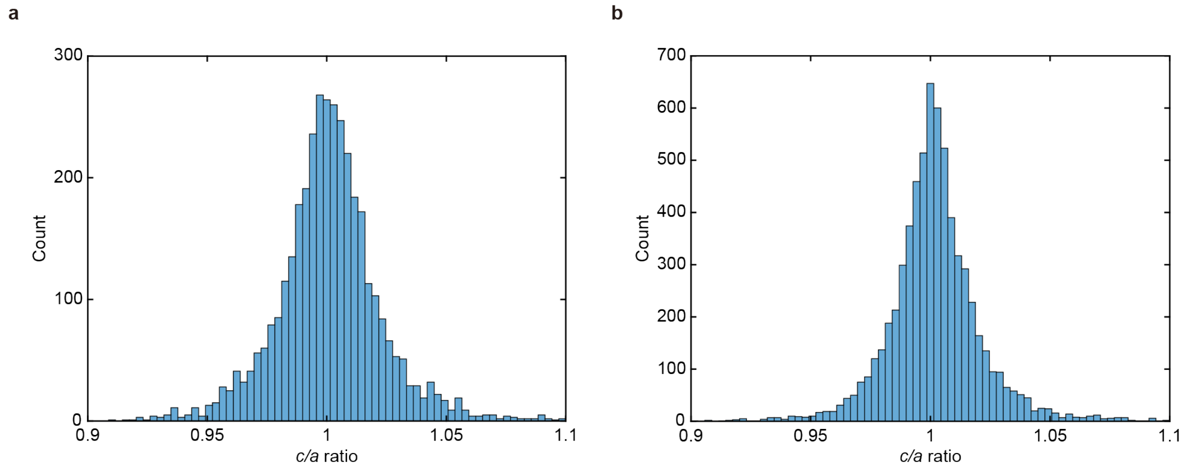

Supplementary Figure 11 | Distribution of the kernel averaged *c/a* ratios. a-b, Histograms of the individual *c/a* ratios for Particle 1 (**a**), and Particle 2 (**b**), respectively. Note that the *c/a* ratio at each atom position is interpolated from the kernel-averaged tetragonality (*c/a*) map. Data of (**a-b**) are provided as a Supplementary Data 1 file.

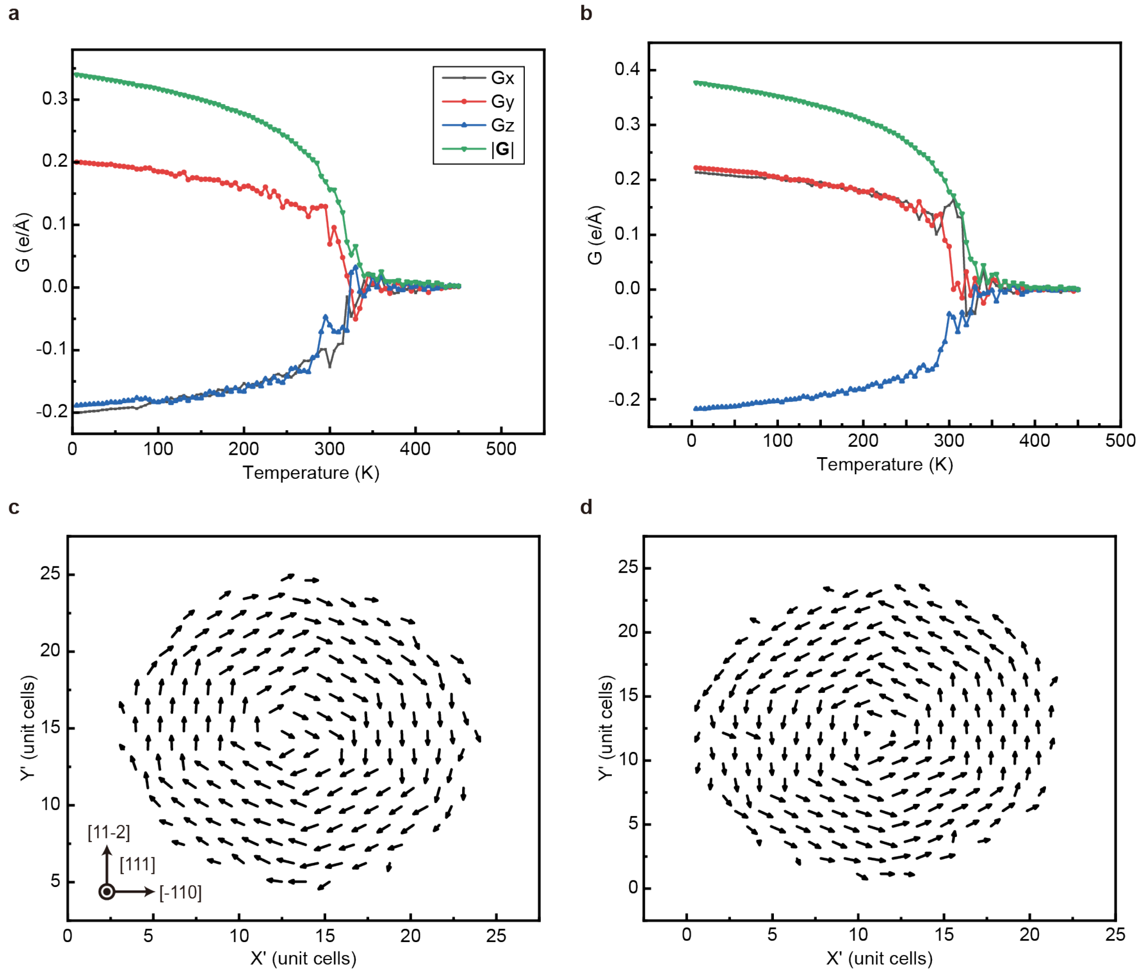

Supplementary Figure 12 | Effective Hamiltonian simulation results for temperature dependence. a, **b**, Simulated *x*, *y*, *z* components of the toroidal moment vector **G** (G_x, G_y, and G_z, respectively) and the absolute value of **G** as a function of temperature for the smaller particle (Particle $\tilde{1}$) (**a**), and larger particle (Particle $\tilde{2}$) (**b**). **c**, **d**, Simulated local 2D dipolar structure maps at 5 K, representing the slices perpendicular to the [111] direction for the smaller (8.8 nm) (**c**) and larger (9.7 nm) (**d**) particles. The slices were obtained at the middle of the nanoparticles. The lattice constant of 5-atom unit cell was 4.01 Å in the simulation.

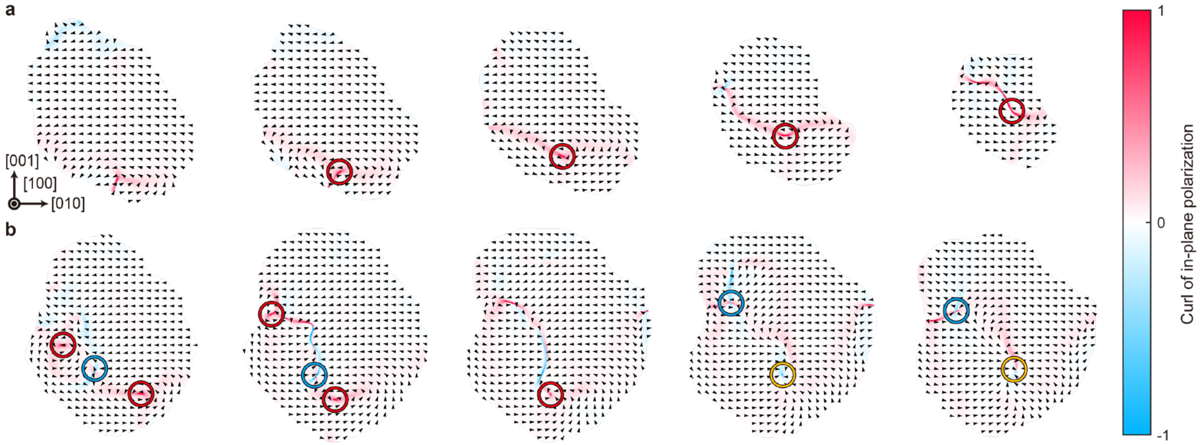

Supplementary Figure 13 | Representative 2D slices through the nanoparticles showing the curl of in-plane polarization. **a**, **b**, Curl of in-plane polarization ($\nabla \times \hat{\boldsymbol{\delta}}_{Ti}$) maps of representative Ti atomic layers sliced along the [100] direction of the Particle 1 (8.8 nm) (**a**), and Particle 2 (10.1 nm) (**b**). The in-plane directions of the Ti displacements are overlaid (black arrows). The curl of the in-plane polarization was calculated as the *x*-component of the curl for the normalized in-plane polarization fields (Methods). Note that the blue and red colors represent the clockwise and counterclockwise rotations, respectively. The existence of vortices (winding number +1, marked with red circles), hedgehog-type structures (winding number +1, marked with yellow circles) and antivortices (winding number −1, marked with blue circles) can be clearly seen. The distance between the colored arrows is 3.75 Å.

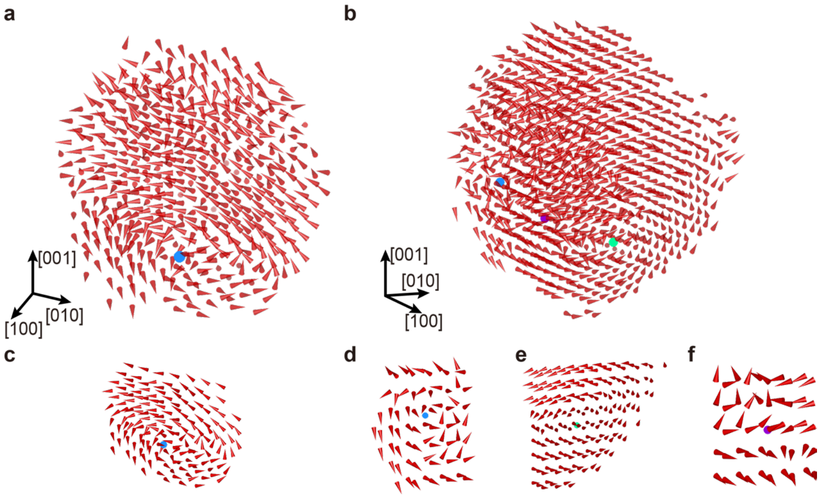

Supplementary Figure 14 | 3D structures of polarization distributions. a-f, 3D polarization configurations of the Particle 1 (**a, c**) and the Particle 2 (**b, d-f**). (**a, b**) show the polarization distributions of the entire nanoparticles. (**c**) and (**d, e**) display the zoomed-in views of the vortex core regions of Particle 1 (marked with a blue circle) and Particle 2 (marked with blue and green circles), respectively. (**f**) displays the zoomed-in view of the antivortex region (marked with a purple circle) found in Particle 2. The orientations of the zoomed-in views are consistent with the orientations of each particle. Note that each polarization vector is represented as a unit vector for visualization purposes.

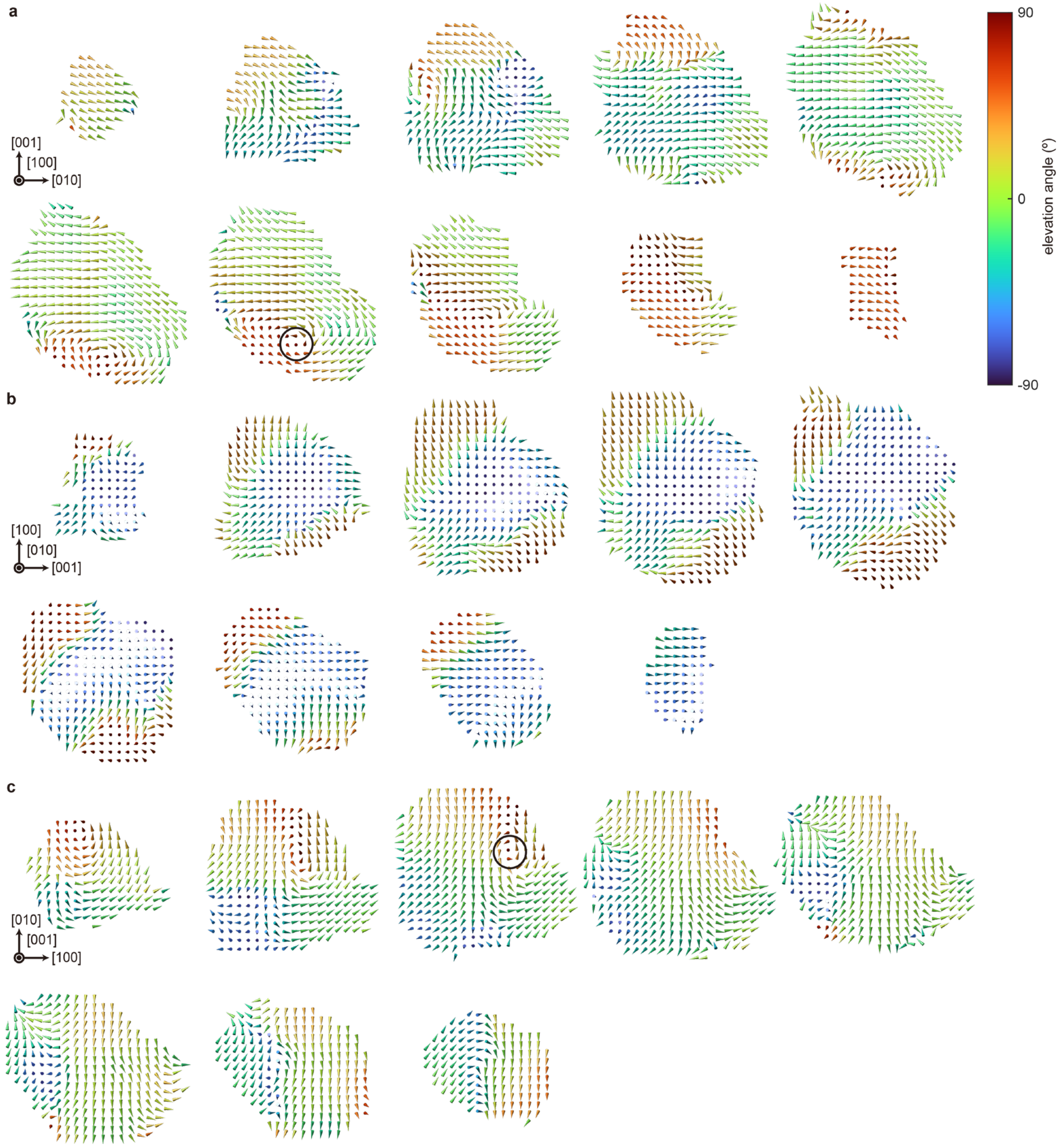

Supplementary Figure 15 | Sliced maps showing the in-plane and out-of-plane polarization configurations for the 8.8 nm $BaTiO_3$ (Particle 1). a-c, 3D Ti atomic displacement maps of representative Ti atomic layers sliced along the [100] (**a**), the [010] (**b**), and the [001] (**c**) directions of the Particle 1. The arrows in the maps indicate the direction of 3D displacement, and their colors reflect the elevation angle between the displacement vector and the plane perpendicular to the [100], [010], and [001] directions for (**a**), (**b**), and (**c**), respectively. A fully red arrow (+90°) points to the [100], [010], and [001] directions, while a fully blue arrow (–90°) points to the [$\bar{1}$00], [0$\bar{1}$0], and [00$\bar{1}$] directions for (**a**), (**b**), and (**c**), respectively. Note that the vortex core (marked with a black circle) found in (**c**) is the same vortex core (marked with a black circle) observed in (**a**). The distance between the colored arrows is 3.75 Å.

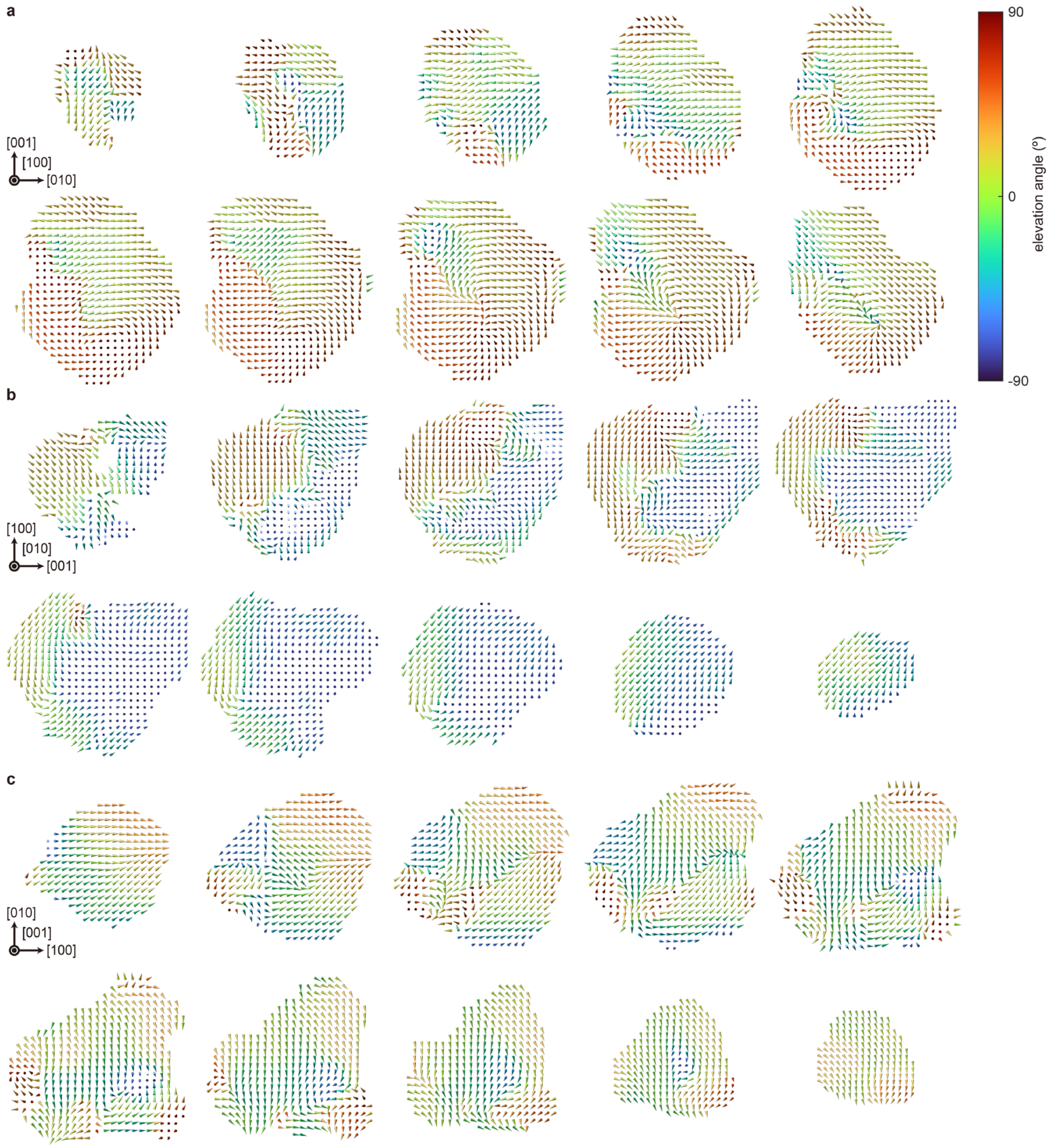

Supplementary Figure 16 | Sliced maps showing the in-plane and out-of-plane polarization configurations for the 10.1 nm $BaTiO_3$ (Particle 2). a-c, 3D Ti atomic displacement maps of representative Ti atomic layers sliced along the [100] (**a**), the [010] (**b**), and the [001] (**c**) directions of the Particle 2. The arrows in the maps indicate the direction of 3D displacement, and their colors reflect the elevation angle between the displacement vector and the plane perpendicular to the [100], [010], and [001] directions for (**a**), (**b**), and (**c**), respectively. A fully red arrow (+90°) points to the [100], [010], and [001] directions, while a fully blue arrow (–90°) points to the [$\bar{1}$00], [0$\bar{1}$0], and [00$\bar{1}$] directions for (**a**), (**b**), and (**c**), respectively. The distance between the colored arrows is 3.75 Å.

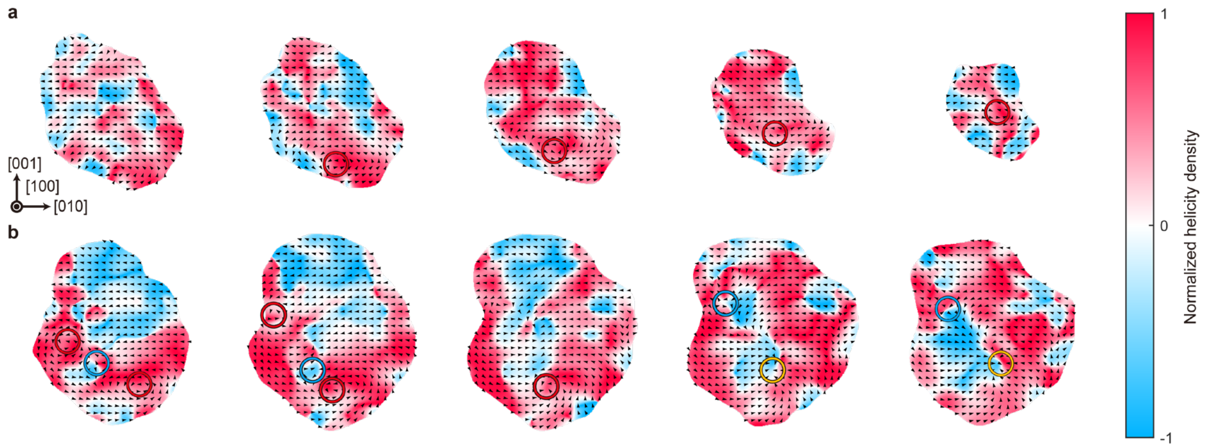

Supplementary Figure 17 | Representative 2D slices through the nanoparticles showing normalized helicity density. a, **b**, Normalized helicity density maps of representative Ti atomic layers along the [100] direction of Particle 1 (8.8 nm) (**a**), and Particle 2 (10.1 nm) (**b**). The in-plane directions of the Ti displacements are overlaid (black arrows). Note that the red (larger than 0) and blue (smaller than 0) colors represent right-handed and left-handed chirality, respectively. The regions of vortices, antivortices, and hedgehog-type structures are marked with red, blue, and yellow circles, respectively. The distance between the arrows is 3.75 Å.

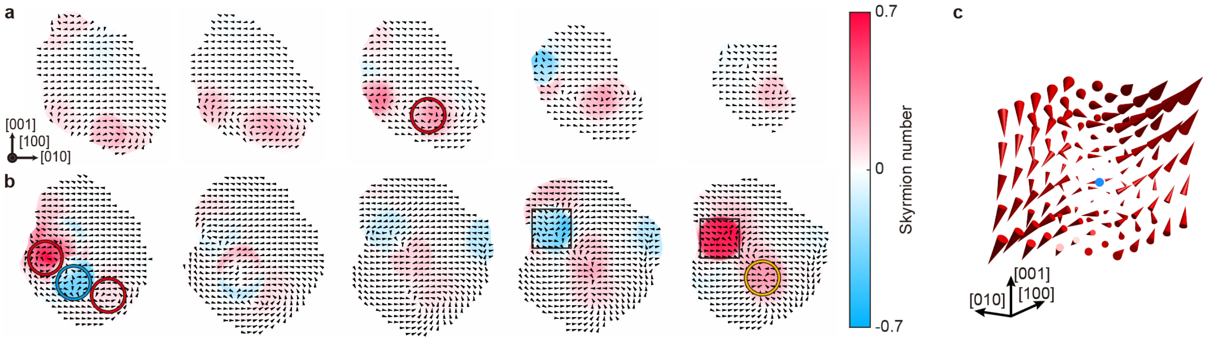

Supplementary Figure 18 | Representative 2D slices through the nanoparticles showing skyrmion numbers. **a**, **b**, Skyrmion number maps of representative Ti atomic layers along the [100] direction of Particle 1 (8.8 nm) (**a**), and Particle 2 (10.1 nm) (**b**). The in-plane directions of the Ti displacements are overlaid (black arrows). Vortices, antivortices, and hedgehog-type structures are marked with red, blue, and yellow circles, respectively. The distance between the arrows is 3.75 Å. The region where the sign of skyrmion number rapidly changes (from –0.5 to 0.6) has been marked with black squares. **c**, The 3D polarization configuration of the volume corresponding to $1.25 \times 1.25 \times 1.25$ Å^3 around the Bloch point (marked with a blue circle) within the region marked with black squares in (**b**).

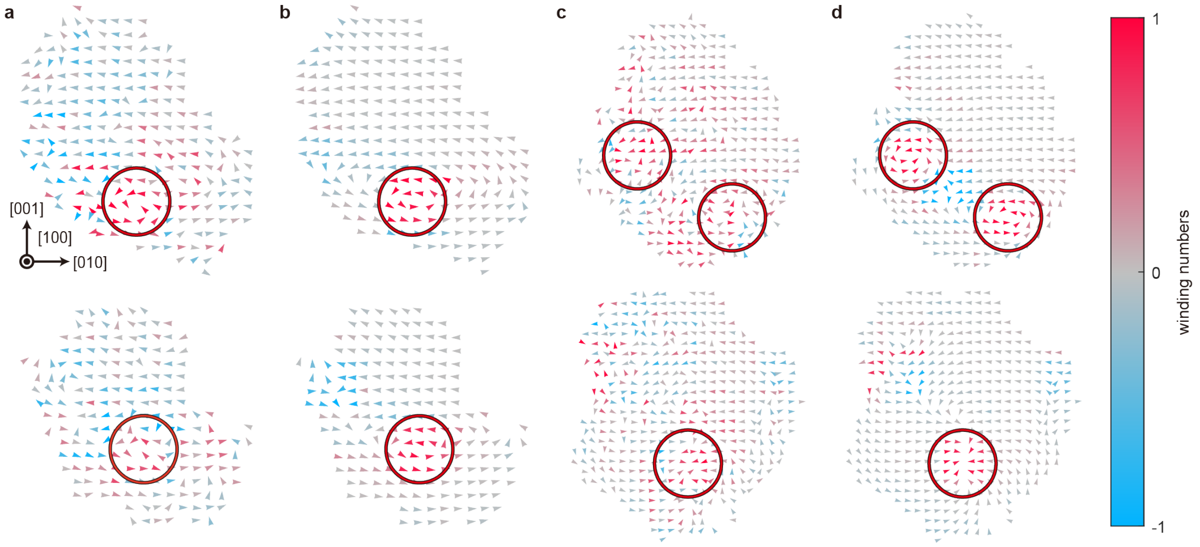

Supplementary Figure 19 | Representative 2D slices through the nanoparticles showing winding numbers. **a**, **b**, Winding number maps of representative Ti atomic layers along the [100] direction of Particle 1 (8.8 nm), before (**a**) and after (**b**) applying a Gaussian kernel. **c**, **d**, Winding number maps of representative Ti atomic layers along the [100] direction of Particle 2 (10.1 nm), before (**c**) and after (**d**) applying a Gaussian kernel. The areas with high winding number values were marked with red circles. Note that in (**b**) and (**d**), the displacement vector at each atom position is interpolated from the kernel-averaged displacement vector map.
