# Supplementary material for: Revealing the three-dimensional arrangement of polar topology in nanoparticles
Source: Nat Commun. 2024 May 8;15:3887. doi: 10.1038/s41467-024-48082-x (PMC11078976; doi:10.1038/s41467-024-48082-x)
Supplement: Supplementary file 3 — Description of Additional Supplementary Files [file 41467_2024_48082_MOESM3_ESM.docx]

**Description of Additional Supplementary Files**

File Name: Supplementary Data 1

Description: This contains the raw data of graphs/histograms within the Supplementary Figures 1, 7, 9, and 11.
